# Comparison of 24-h Urine Protein, Urine Albumin-to-Creatinine Ratio, and Protein-to-Creatinine Ratio in IgA Nephropathy

**DOI:** 10.3389/fmed.2022.809245

**Published:** 2022-02-28

**Authors:** Guizhen Yu, Jun Cheng, Heng Li, Xiayu Li, Jianghua Chen

**Affiliations:** ^1^Kidney Disease Center, The First Affiliated Hospital, College of Medicine, Zhejiang University, Hangzhou, China; ^2^Key Laboratory of Kidney Disease Prevention and Control Technology, Zhejiang University, Hangzhou, China; ^3^National Key Clinical Department of Kidney Diseases, Zhejiang University, Hangzhou, China; ^4^Institute of Nephrology, Zhejiang University, Hangzhou, China; ^5^The Third Grade Laboratory Under the National State, Administration of Traditional Chinese Medicine, Hangzhou, China

**Keywords:** IgA nephropathy, protein-to-creatinine ratio (PCR), albumin-to-creatinine ratio (ACR), 24-h urine protein excretion (24-h UP), kidney disease progression

## Abstract

**Background:**

Proteinuria is a strong risk factor for renal outcomes in IgA nephropathy. Random urine protein-to-creatinine ratio (PCR), random albumin-to-creatinine ratio (ACR), and 24-h urine protein excretion (24-h UP) have been widely used in clinical practice. However, the measurement which is the best predictor of long-term renal outcomes remains controversial. This study aimed to compare the three measurements in IgA nephropathy.

**Methods:**

We conducted a retrospective study of 766 patients with IgA nephropathy. The associations among baseline ACR, PCR, and 24-h UP with chronic kidney disease (CKD) progression event, defined as 50% estimated glomerular filtration rate (eGFR) decline or end stage kidney disease (ESKD), were tested and compared.

**Results:**

In this study, ACR, PCR, and 24-h UP showed high correlation (*r* = 0.671–0.847, *P* < 0.001). After a median follow-up of 29.88 (14.65–51.65) months, 51 (6.66%) patients reached the CKD progression event. In univariate analysis, ACR performed better in predicting the prognosis of IgA nephropathy, with a higher area under the receiver operating curve (ROC) curve than PCR and 24-h UP. After adjustment for traditional risk factors, ACR was most associated with composite CKD progression event [per log-transformed ACR, hazard ratio (HR): 2.82; 95% (95% CI): 1.31–6.08; *P* = 0.008].

**Conclusions:**

In IgA nephropathy, ACR, PCR, and 24-h UP had a high correlation. ACR performed better in predicting the prognosis of IgA nephropathy.

## Introduction

IgA nephropathy is the most common primary glomerulonephritis worldwide and the main cause of end-stage kidney disease (ESKD) (1). IgA nephropathy presents a variable clinical course ranging from asymptomatic hematuria to rapidly progressive kidney failure (2, 3). Proteinuria is one of the symptoms and has been established to be a robust predictor of outcome in IgA nephropathy.

Three measurements are used for proteinuria evaluation in clinical practice: 24-h urine protein excretion (24-h UP), random urine protein-to-creatinine ratio (PCR), and random albumin-to-creatinine ratio (ACR). Despite being considered the “gold-standard” in therapeutic guidelines, 24-h UP is cumbersome to perform, inconvenient for patients, and subject to sample collection variability. ACR and PCR in random spot urine measurements are commonly used. Numerous studies have investigated the quantitative assessment of proteinuria by using ACR and PCR in kidney disease and other diseases involving proteinuria (4–6). However, studies have shown conflicting results regarding the correlation among ACR, PCR, and 24-h UP. Some studies have indicated poor correlations among ACR, PCR, and 24-h UP measurements (7), whereas others have found a high degree of correlation among these measurements and suggested that they are equivalent predictors of renal outcomes (8–11). Therefore, whether PCR and ACR could replace the traditional 24-h UP and which measurement of proteinuria is most associated with long-term renal outcome remain unclear.

In this study, we aimed to evaluate the relationships among ACR, PCR, and 24-h UP in IgA nephropathy, and examine the efficacy of the three measurements in predicting the prognosis in IgA nephropathy.

## Methods

### Study Population

In this retrospective study, a total of 766 patients with biopsy-proven IgA nephropathy, diagnosed between 2002 and 2019, were selected from The First Affiliated Hospital, Zhejiang University School of Medicine. All participants were followed up for more than 6 months and had complete clinical data. Patients with secondary causes of IgA deposition, such as liver cirrhosis, systemic lupus, and IgA vasculitis, were excluded. Minimal change disease was excluded in order to ensure the homogeneity of this cohort (12).

The study was approved by the local ethics committees, and written informed consent was obtained from all participants.

### Information of Clinical Manifestations

In our center, ACR, PCR, and 24-h UP were collected within the same time frame. Random spot urine measurements were used for the detection of ACR and PCR. ACR and PCR were calculated as spot urinary albumin concentration and urinary protein concentration divided by urinary creatinine concentration, respectively (9). In addition, 24-h urine samples were collected for 24-h UP analysis.

Information of ACR, PCR, and 24-h UP, as well as other clinical data, including sex, age, blood pressure, and serum creatinine at the time of kidney biopsy, were collected from medical records. The estimated glomerular filtration rate (eGFR) was calculated with the Chronic Kidney Disease Epidemiology Collaboration (CKD-EPI) equation (13). Hypertension was defined as systolic BP ≥140 mmHg and/or diastolic BP ≥90 mmHg. Mean arterial pressure (MAP) was calculated as the sum of diastolic BP plus one-third of the pulse pressure. Nephrotic syndrome was defined as a 24-h urinary protein level exceeding 3.5 g/d. Kidney failure was defined as eGFR <15 ml/min/1.73 m^2^, dialysis, or transplantation. The composite kidney disease progression event was defined as kidney failure and/or 50% eGFR decline.

Whether long-term outcome differs in IgA nephropathy patients with proteinuria between 0.5 and 1.0 g/d compared to <0.5 g/d remains unclear (14). Therefore, we divided patients into three groups according to the proteinuria levels in ACR (g/g), PCR (g/g), and 24-h UP (g/d), as group 1: ≤ 0.5; group 2: 0.5–1.0; and group 3: ≥1.0.

### Statistical Analyses

Normally distributed data were expressed as mean ± SD and compared with a *t*-test. Non-normally distributed variables were summarized as medians or interquartile ranges and compared with Mann–Whitney U test. Qualitative variables were described in ratios or percentages and analyzed with a Chi-squared test. The Cox regression model was used to analyze the relationship between parameters and kidney disease progression events. The receiver operating curve (ROC) was used to compare the performance of ACR, PCR, and 24-h UP in predicting the prognosis of IgA nephropathy. The cumulative incidence function method was used to evaluate the association between proteinuria and kidney disease progression events. Statistical analysis was performed in SPSS 24.0, Stata software 14.0, and GraphPad Prism 8.0. A value of *P* < 0.05 was considered significantly different.

## Results

### Clinical Characteristics of IgA Nephropathy

A total of 766 patients with IgA nephropathy with a median follow-up of 29.88 (14.65–51.65) months were included in this study. The baseline characteristics of the participants are presented in Table 1. The cohort included 391 (51.04%) men with a mean age of 38.05 ± 11.75 years. At the time of renal biopsy, the MAP level was 94.28 ± 13.66 mmHg, and the median eGFR was 83.70 ± 29.19 ml/min/1.73 m^2^. The baseline 24-h UP, PCR, and ACR were 0.99 (0.49–2.05) g/d, 0.93 (0.49–1.82) g/g, and 0.55 (0.29–1.21) g/g, respectively. In total, 51 (6.66%) patients reached composite kidney disease progression events, including 37 (4.83%) kidney failure events.

**Table 1:** Baseline clinical characteristics of patients with IgA nephropathy.

**Characteristic**	**Value, *n* = 766**
**Baseline**	
Male sex	391 (51.04)
Age, y	38.05 ± 11.75
MAP, mmHg	94.28 ± 13.66
Serum albumin, g/L	39.19 ± 6.07
24 Proteinuria, g/d	0.99 (0.49–2.05)
PCR, g/g	0.93 (0.49–1.82)
ACR, g/g	0.55(0.29–1.21)
eGFR, mL/min/1.73 m^2^	83.70 ± 29.19
BMI (kg/m^2^)	22.93 ± 3.30
**CKD stages**, ***n*** **(%)**	
1	354 (46.21)
2	219 (28.59)
3	172 (22.45)
4	21 (2.74)
**Oxford classification**, ***n*** **(%)**	
M1	111 (14.49)
E1	52 (6.79)
S1	457 (59.66)
T1–T2	91 (11.88)
C1–C2	289 (37.73)
**Follow-up and outcome**	
Follow-up duration, mo	29.88 (14.65–51.65)
50% eGFR decline, %	43 (5.61)
Kidney failure, %	37 (4.83)
Composite outcome, %	51 (6.66)

*Values for continuous variables are expressed as mean ± SD or median [interquartile range]; count (percentage) is used for categorical variables. The composite outcome was defined as a 50% eGFR decline or kidney failure*.

### Correlation Among ACR, PCR, and 24-h UP

**Figure 1:**
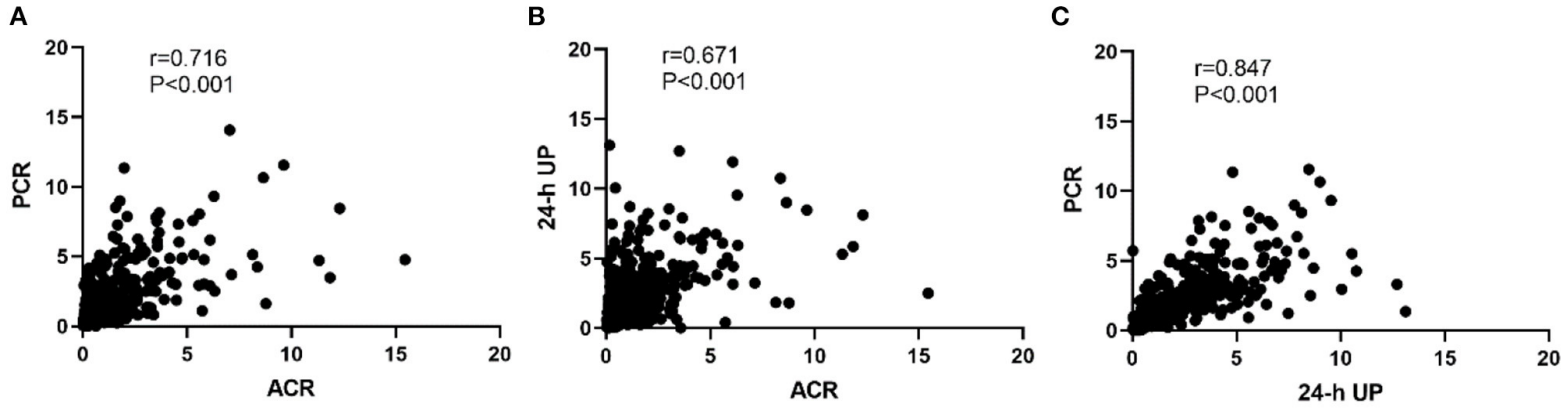
Correlation among PCR, ACR, and 24-h UP. **(A)** The correlation between PCR and ACR. **(B)** The correlation between ACR and 24-h UP. **(C)** The correlation between PCR and 24-h UP. The conversion coefficients between PCR and ACR, 24-h UP and ACR were 0.84 and 0.96, respectively, which means that PCR multiplied by 0.84 equals the value of ACR, and 24-h UP multiplied by 0.96 equals the value of ACR.

As shown in Figure 1, a high degree of correlation was observed among ACR, PCR, and 24-h UP. PCR and 24-UP showed the best correlation (*r* = 0.847, *P* < 0.001), followed by PCR and ACR (*r* = 0.716, *P* < 0.001), and the correlation between ACR and 24-h UP was lowest (*r* = 0.671, *P* < 0.001). The conversion coefficients between PCR and ACR, and 24-h UP and ACR were 0.84 and 0.96, respectively, which means that PCR multiplied by 0.84 equals the value of ACR, and 24-h UP multiplied by 0.96 equals the value of ACR. Because nephrotic syndrome is uncommon in IgA nephropathy, we analyzed patients with 24-h UP <3.5 g/d. The correlations between ACR and 24-h UP (*r* = 0.629, *P* < 0.001), PCR and 24-h UP (*r* = 0.795, *P* < 0.001), and PCR and ACR (*r* = 0.673, *P* < 0.001) were better than those with 24-h UP ≤ 3.5 g/d.

This study included 96 (12.53%) patients with IgA nephropathy with nephrotic syndrome. We also analyzed the correlations among serum albumin, ACR, PCR, and 24-h UP in patients with higher ranges of proteinuria. A negative correlation between albumin and 24-h UP was observed (*r* = −0.489, *P* < 0.001). The Spearman correlation coefficients between albumin and ACR, and albumin and PCR were −0.329 and −0.365, respectively (Supplementary Figure S1).

### ACR, PCR, 24-h UP, and Disease Severity and Progression in IgA Nephropathy

We analyzed the correlation between ACR, PCR, and 24-h UP at the time of renal biopsy with the severity of IgA nephropathy, and found these three measurements all correlated with chronic kidney disease (CKD) stage, hypertension, and Oxford E and T lesions. Patients with higher levels of 24-UP, PCR, and ACR showed severe CKD stage, hypertension, and Oxford E and T lesions (Table 2).

**Table 2:** Correlation between 24-h urinary protein excretion (24-h UP), PCR, and ACR and severity of IgA nephropathy.

**Characteristics**	**24-h UP**		**PCR**		**ACR**	
	**g/d**	***p***	**g/g**	***p***	**g/g**	***p***
CKD stage		<0.001		<0.001		<0.001
1	0.82 (0.43, 1.60)		0.83 (0.43, 1.53)		0.46 (0.23, 0.98)	
2	1.01 (0.49, 2.08)		0.84 (0.47, 1 .56)		0.49 (0.29, 0.95)	
3	1.48 (0.60, 3.15)		1.24 (0.69, 2.54)		0.83 (0.39, 1.96)	
4	3.15 (1.58, 4.31)		2.90 (1.47, 4.06)		1.61 (0.87, 3.01)	
Hypertension		<0.001		<0.001		<0.001
With	1.54 (0.68, 2.72)		1.310 (0.60, 2.15)		0.78 (0.41, 1.45)	
Without	0.86 (0.46, 1.71)		0.84 (0.43, 1.63)		0.47 (0.27, 1.08)	
Oxford classification						
M lesion		0.092		0.055		0.113
1	1.10 (0.58, 2.37)		1.00 (0.64, 2.05)		0.63 (0.31, 1.40)	
0	0.97 (0.48, 2.00)		0.92 (0.47, 1.80)		0.53 (0.28, 1.20)	
E lesion		<0.001		<0.001		<0.001
1	2.11 (0.82, 5.67)		2.24 (0.84, 4.21)		1.11 (0.39, 2.41)	
0	0.95 (0.48, 1.91)		0.89 (0.47, 1.70)		0.53 (0.28, 1.14)	
S lesion		0.667		0.403		0.401
1	0.99 (0.51, 1.91)		0.94 (0.54, 1.79)		0.51 (0.31, 1.06)	
0	0.97 (0.46, 2.26)		0.88 (0.42, 1.91)		0.61 (0.25, 1.41)	
T lesion		<0.001		<0.001		<0.001
1 or 2	1.93 (0.67, 3.71)		1.70 (0.73, 2.79)		0.97 (0.42, 2.07)	
0	0.94 (0.48, 1.85)		0.88 (0.47, 1.66)		0.51 (0.28, 1.11)	

In unadjusted Cox models, 24-h UP, ACR, and PCR were associated with composite kidney disease progression events. The value of ACR was highest [24-h UP: hazard ratio (HR), 3.70; 95% CI: 1.96–6.99; *P* < 0.001; PCR: HR, 3.65; 95% CI: 1.94–6.88; *P* < 0.001; ACR: HR, 3.92; 95% CI: 2.22–6.94; *P* < 0.001]. After adjustment for the traditional risk factors of age, sex, MAP, eGFR, and Oxford classification (MEST-C scores), ACR had the highest HR value, compared with PCR and 24-h UP (ACR: HR, 2.82; 95% CI: 1.31–6.08; 24-h UP: HR, 2.75; 95% CI: 1.17–6.47; PCR: HR, 2.50; 95% CI:1.06–5.91) (Table 3).

**Table 3:** Association of 24-h urinary protein excretion and PCR with composite kidney disease progression.

	**Hazard ration, 95% confidence interval and** ***P*****-value**			
	**Unadjusted**	**Model 1**	**Model 2**	**Model 3**
Lg (24-h UP)	3.70 (1.96–6.99) <0.001	3.54 (1.85–6.76) <0.001	1.71 (0.78–3.73) 0.178	2.75 (1.17–6.47) 0.021
Lg (PCR)	3.65 (1.94–6.88) <0.001	3.36 (1.73–6.49) <0.001	1.68 (0.75–3.72) 0.205	2.50 (1.06–5.91) 0.037
Lg (ACR)	3.92 (2.22–6.94) <0.001	3.69 (2.05–6.66) <0.001	2.33 (1.08–5.05) 0.032	2.82 (1.31–6.08) 0.008

*A CKD progression event was defined as 50% eGFR or kidney failure*.

*Model 1 was adjusted for sex and age; sex is expressed as a dichotomous variable*.

*Model 2 was adjusted for covariates in Model 1 plus mean arterial pressure and baseline eGFR*.

*Model 3 was adjusted for covariates in model 2 plus Oxford classification (MEST-C scores)*.

**Figure 2:**
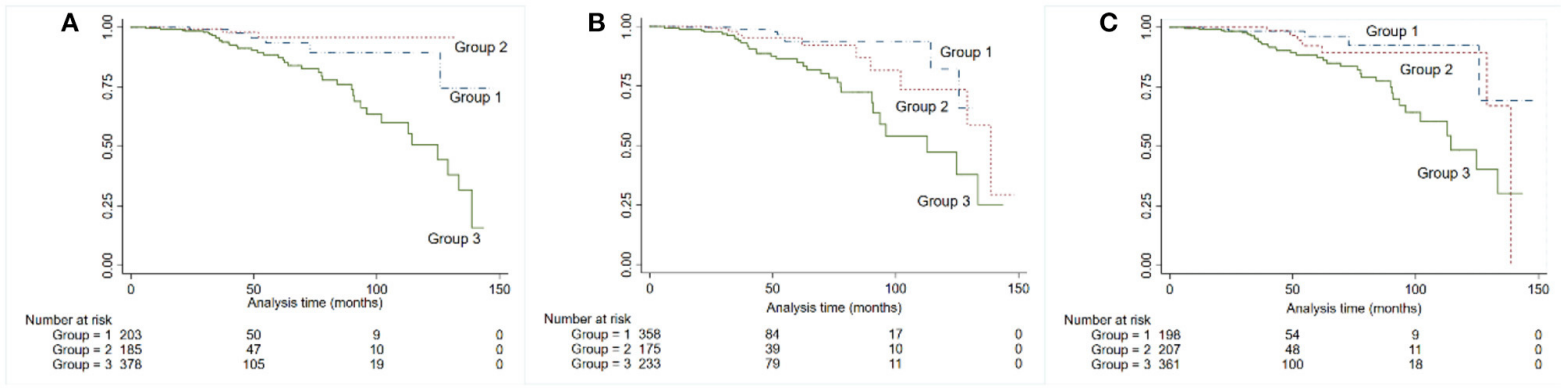
Kaplan-Meier kidney survival curves for patients with IgA nephropathy, according to 24-h urine protein **(A)**, albumin-to-creatinine ratio **(B)**, and PCR **(C)**. Time zero was the time of kidney biopsy. The divisions among the three groups were based on the levels of proteinuria: group 1: ≤ 0.5, group 2: 0.5–1.0, group 3: ≥1.0.

After dividing patients into three groups according to the proteinuria levels, we found that in ACR, patients with proteinuria <0.5 had the best prognosis, although the difference was not statistically significant (*P* = 0.238), and this was followed by proteinuria 0.5–1.0, while patients with proteinuria ≥1.0 had the poorest prognosis. In terms of PCR and ACR, proteinuria <0.5 performed similar to 0.5–1.0 in long-term outcome survival curve (PCR: *P* = 0.621; 24-h UP: *P* = 0.434) (Figure 2).

**Figure 3:**
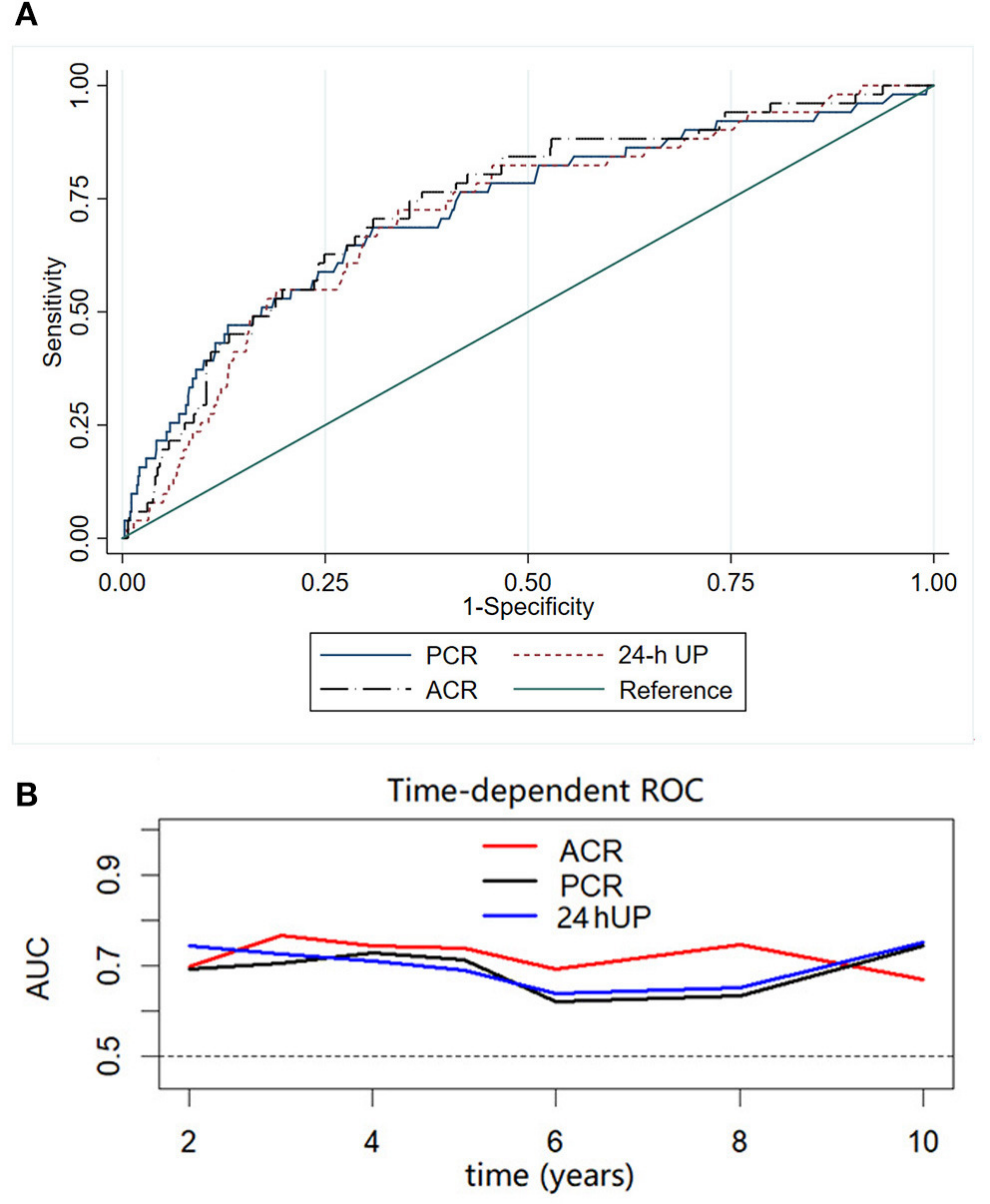
Receiver operating characteristic (ROC) **(A)** and time-dependent ROC **(B)** curves with the composite outcome as the status variable. The AUCs were compared among ACR, PCR, and 24-h UP using ROC and time-dependent ROC curves. Among these indicators, ACR had the highest AUC.

To compare the performance of ACR, PCR, and 24-h UP in predicting renal outcome, we used ROC curve analysis, and the area under the ROC curves (AUC) was calculated. The AUC values and 95% CI of 24-h UP, ACR, and PCR were 0.71 (0.64–0.79), 0.74 (0.67–0.81), and 0.72 (0.65–0.80), respectively. Among these indicators, ACR had the highest AUC (Figure 3A). Results were consistent when time-dependent ROC was used to compare ACR, PCR, and 24-h UP (Figure 3B).

Furthermore, we evaluated the effects of ACR, PCR, 24-h UP, and serum albumin levels on predicting IgA nephropathy progression in patients with nephrotic syndrome. We found that the AUC values were 0.60 (0.47–0.73), 0.62 (0.47–0.77), 0.44 (0.28–0.60), and 0.55 (0.42–0.69), respectively (Supplementary Figure S2).

## Discussion

Proteinuria is a strong risk factor in predicting the prognosis of IgA nephropathy. ACR and PCR in random spot urine measurements and 24-h UP are commonly used to evaluate proteinuria in clinical practice. However, whether PCR or ACR might replace the traditional 24-h UP examination, and which measurement of proteinuria is the best predictor of long-term renal outcomes in IgA nephropathy remain unknown. In this study of 766 patients with IgA nephropathy, we found that ACR and PCR showed a high correlation with 24-h UP. After stratifying analyses by proteinuria at 3.5 g/d, we found that the correlations among ACR, PCR, and 24-h UP were similar to those in the overall study population. With respect to prediction of long-term renal outcome, defined as 50% eGFR decline or renal failure, ACR had the best correlation, compared with PCR and 24-h UP. Another key finding was that, in patients with nephrotic syndrome, serum albumin was found to be a tool for assessing the extent of proteinuria.

The “gold standard” method for evaluating proteinuria is quantifying the protein concentration in the urine for 24 h (8). However, 24-h UP evaluated with 24-h urine collection is troublesome and time consuming for patients, and sometimes involves errors that can affect the accuracy of the results (15–18). PCR and ACR in random spot urine measurements are two simple and fast methods for quantitative assessment of urine protein excretion, which are commonly used in clinical practice. However, several reports comparing PCR, ACR, and 24-h UP have yielded conflicting results. Some studies have shown that PCR and ACR have a poorer correlation in lower ranges of proteinuria (19). Multiple studies have indicated that ACR is superior to PCR in the consistency, sensitivity, and quality of the approach for early detection and management of CKD (20, 21). Jinn-Yuh Guh has reported that ACR performs better in patients with diabetes, whereas PCR performs better in non-diabetic patients (22). In contrast, a study from the UK has demonstrated that PCR is more sensitive than ACR in patients with proteinuria <0.5 g/d and <1.0 g/d (23). Many studies have established that ACR and PCR display a strong linear correlation with 24-h UP (21, 24, 25). In a UK cohort of 1,676 patients with CKD over 3.5 years of follow-up, ACR and PCR have been found to perform as well as 24-h UP in predicting renal outcome in patients with CKD; however, the PCR and ACR were derived from aliquots of 24-h urine samples rather than random spot urine measurements in that study (11). Given the conflicting results, the recommendations vary among guidelines. In 2012, the Kidney Disease: Improving Global Outcomes (KDIGO) guidelines recommended ACR over PCR measurement, partly because of sample-to-sample variation, the composition of urine protein, and inter-laboratory variation in PCR measurement. Interestingly, despite the limitations of PCR measurement, PCR has been found to be similar to ACR in its association with complications in CKD (26). The recently published KDIGO guidelines recommend that 24-h UP or PCR should be measured when therapeutic decisions about using high-risk medications are being made on small changes in proteinuria. PCR from an intended 24-h UP collection that is at least 50% complete has been shown to accurately reflect 24-h UP (27, 28). Some recommendations have advocated using ACR to replace PCR measurement (21, 29, 30), whereas others have suggested continuing the use of PCR.

In this much larger sample study with 766 patients with IgA nephropathy, we confirmed that PCR, ACR, and 24-h UP displayed a strong linear relationship. Compared with PCR and 24-h UP, ACR performed best in predicting the long-term renal outcomes, defined as a 50% eGFR decline or ESKD in IgA nephropathy. To our knowledge, only one prior study has compared different measurements of proteinuria in IgA nephropathy. In that study, similarly to our findings, ACR, PCR, and 24-h UP showed a good correlation, and all three measurements of proteinuria were associated with poor renal outcome, defined as 30% eGFR decline, ESKD, or death in a cohort study with 438 patients with IgA nephropathy (21). Our study confirmed the previous results in a larger sample with 766 patients with IgA nephropathy and defined a robust renal outcome defined as 50% eGFR decline or renal failure. Furthermore, we found that in patients with nephrotic syndrome, the serum albumin levels were negatively correlated with proteinuria, which is in agreement with a prior hypothesis that monitoring serum albumin levels in nephrotic patients might also be a valuable tool to indirectly assess the extent of proteinuria (27). However, nephrotic syndrome is uncommon in IgA nephropathy and was found in only 96 (12.14%) patients in our center. Therefore, further confirmation in larger samples is needed.

Whether long-term outcome differs in patients with proteinuria between 0.5 and 1.0 g/d compared with <0.5 g/d remains unknown (14). In our study, patients with proteinuria <0.5 g/d had better prognoses than those with proteinuria between 0.5 and 1.0 g/d only in proteinuria measurement of ACR; however, for PCR and 24-h UP, patients with proteinuria <0.5 g and 0.5–1.0 g had similar prognosis. A recent study has reported similar HR values for risk of ESRD for proteinuria <0.5 g/d and 0.51–1.0 g/d in 921 participants with IgA nephropathy, which is in agreement with our findings (31). Whether lowering proteinuria <0.5 g/d is renoprotective still need further confirmation.

The strength of this study includes the large sample size, including many renal outcome events observed in IgA nephropathy. However, this study also has several limitations. First, our patients included only those with IgA nephropathy; thus, we cannot extrapolate our findings to other patients with CKD. Second, all patients in our study were from a single center and were from a single ethnic group, and more than 90% of patients received steroids, renin angiotensin aldosterone system inhibitors (RAASis), and other immunosuppressant therapy during follow-up, and therefore, we did not include the use of immunosuppressant and RAASi in the analysis. According to the recently published 2021 KDIGO Guideline, rapidly progressive IgA nephropathy was defined as 50% eGFR decline within 3 months. However, in our study, all participants were followed up for more than 6 months, and there were no rapidly progressive IgA nephropathy patients. This is another limitation in our study (32). Thus, the results still need further confirmation in a more diverse cohort.

In conclusion, in this study, we demonstrated that ACR, PCR, and 24-h UP displayed a good correlation. ACR and PCR were comparable to 24-h UP in predicting the prognosis of IgA nephropathy.

## Data Availability Statement

The original contributions presented in the study are included in the article/Supplementary Material, further inquiries can be directed to the corresponding author/s.

## Author Contributions

JCheng and JChen are the guarantors of the integrity of the entire study and contributed to the study concepts and study design. GY contributed to data acquisition and interpretation, clinical studies, and manuscript preparation. HL and XL contributed to manuscript editing. JChen contributed to the manuscript review. All authors have contributed significantly. All authors contributed to the article and approved the submitted version.

## Funding

This study was supported by grant LY19H050007 from Zhejiang Natural Science Foundation and grant 2016KYA087 Zhejiang Medical and Health Science and Technology Project.

## Conflict of Interest

The authors declare that the research was conducted in the absence of any commercial or financial relationships that could be construed as a potential conflict of interest.

## Publisher's Note

All claims expressed in this article are solely those of the authors and do not necessarily represent those of their affiliated organizations, or those of the publisher, the editors and the reviewers. Any product that may be evaluated in this article, or claim that may be made by its manufacturer, is not guaranteed or endorsed by the publisher.
