# Supplementary material for: Comparison of 24-h Urine Protein, Urine Albumin-to-Creatinine Ratio, and Protein-to-Creatinine Ratio in IgA Nephropathy
Source: Front Med (Lausanne). 2022 Feb 28;9:809245. doi: 10.3389/fmed.2022.809245 (PMC8918683; doi:10.3389/fmed.2022.809245)
Supplement: Supplementary file 1 [file Data_Sheet_1.docx]

**Supplementary files**

**Figure S1.** **Correlations between 24-h UP and serum albumin (Alb), ACR and serum albumin (Alb), and PCR and serum albumin (Alb).** (A) The correlation between 24-h UP and Alb. (B) The correlation between ACR and Alb. (C) The correlation between PCR and Alb.

**Figure S2. Receiver operating characteristic curves with composite outcome as the status variable in patients with nephrotic syndrome.** The AUCs and 95% CIs of ACR and PCR, 24-h UP and serum albumin (Alb) were 0.60 (0.47–0.73), 0.62 (0.47–0.77), 0.44 (0.28–0.60) and 0.55 (0.42–0.69), respectively.


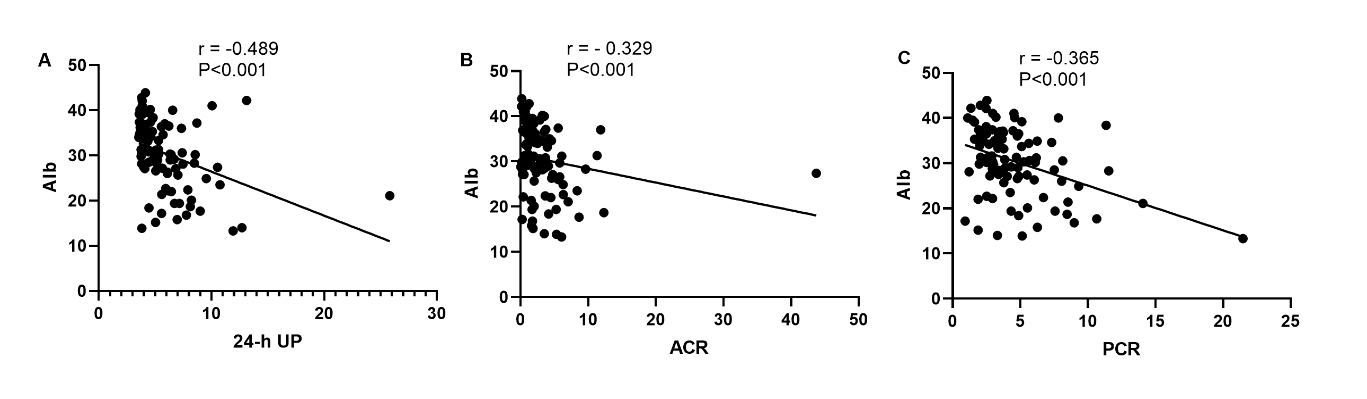


**Figure S1.** **Correlations between 24-h UP and serum albumin (Alb), ACR and serum albumin (Alb), and PCR and serum albumin (Alb).** (A) The correlation between 24-h UP and Alb. (B) The correlation between ACR and Alb. (C) The correlation between PCR and Alb.


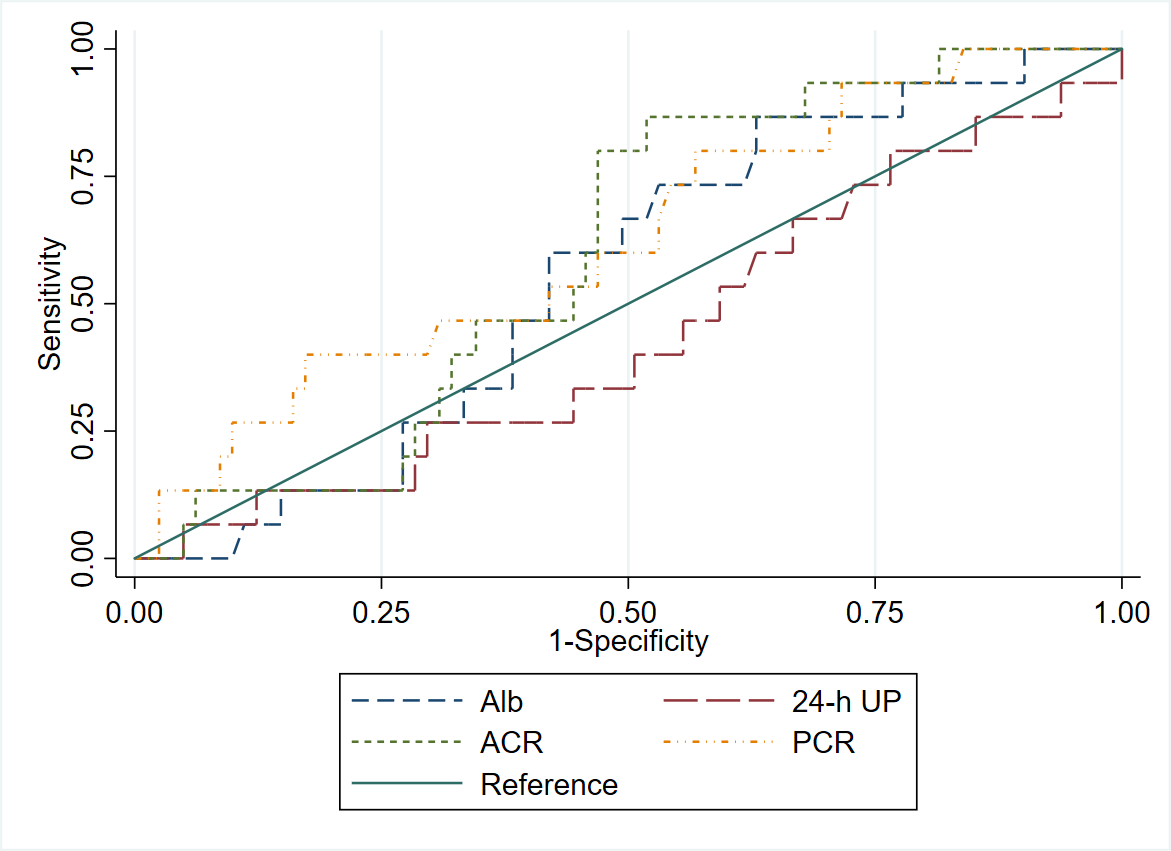


**Figure S2. Receiver operating characteristic curves with composite outcome as the status variable in patients with nephrotic syndrome.** The AUCs and 95% CIs of ACR and PCR, 24-h UP and serum albumin (Alb) were 0.60 (0.47–0.73), 0.62 (0.47–0.77), 0.44 (0.28–0.60) and 0.55 (0.42–0.69), respectively.
